# 肺癌合并肌炎1例临床分析

**DOI:** 10.3779/j.issn.1009-3419.2012.08.09

**Published:** 2012-08-20

**Authors:** 红宇 吴, 宇 刘, 兆瑛 盛

**Affiliations:** 200433 上海，上海市肺科医院放疗科 Department of Radiotherapy, Shanghai Pulmonary Disease Hospital, Shanghai 200433, China

肺癌是最严重的肺部病变，早期及时治疗可以有治愈的希望。发现早期肺癌尤为重要，肺癌早期症状除外咯血、胸痛、咳嗽等肺部症状以外，多发性肌炎等肺外症状应该引起我们的重视，因为肌炎等亦为肺癌的早期症状，据统计85%先于肺癌典型症状出现，表现为渐进性周身无力、食欲减退、加重时可行走困难、卧床难起，其它表现还有无明显原因的声音嘶哑伴气喘、一侧颈部明显浮肿、一侧眼裂变、眼睑下垂等。肺癌的诊治关键在于早发现、早诊断、早治疗。中老年人，尤其是长期吸烟者，如出现上述可疑症状，千万不可掉以轻心，应及时去医院作胸部X线照片或CT等检查，排除肺癌的可能。当然约有15%肺癌患者早期可完全没有症状。

## 1 临床资料

患者，女，52岁，2010年8月气管镜确诊左肺腺癌，并于同年9月-12月行泰素、顺铂化疗，但左颈部淋巴结呈进行性增大，2011年3月胸CT（图 1）提示左肺下叶肿块纵隔肺门淋巴结增大为行放疗收住我科。入院时常规检查乳酸脱氢酶（lactate dehydrogenase, LDH）346 IU/L，肌酸激酶（creatine kinase, CK）1, 169 IU/L，肌酸激酶同工酶（CK-MB）41 IU/L。因心肌酶谱明显高于正常引起临床医生重视但检查后患者临床无心前区疼痛，心电图检查无异常，予查心肌损伤肌钙蛋白（-），肌红蛋白弱阳性，CK-MB（+），脑钠尿肽（brain natriuretic peptide, BNP）5.0 pg/mL，故不考虑心脏疾患致心肌酶升高，后复查LDH 381 IU/L、CK 1, 716 IU/L、CK-MB 63I U/L。结果：追问病史患者诉平日有活动后乏力，行走一段时间后即会感觉无力，给予查肌电图提示肌炎表现，于是临床诊断肺癌合并肌炎，后于外院行肌肉活检病理确诊肌炎（切片见图 2），治疗方法为强的松1 mg/kg口服同时给予胸部颈部肿瘤灶放射治疗，治疗过程中随访心肌酶谱参数下降。

**1:**
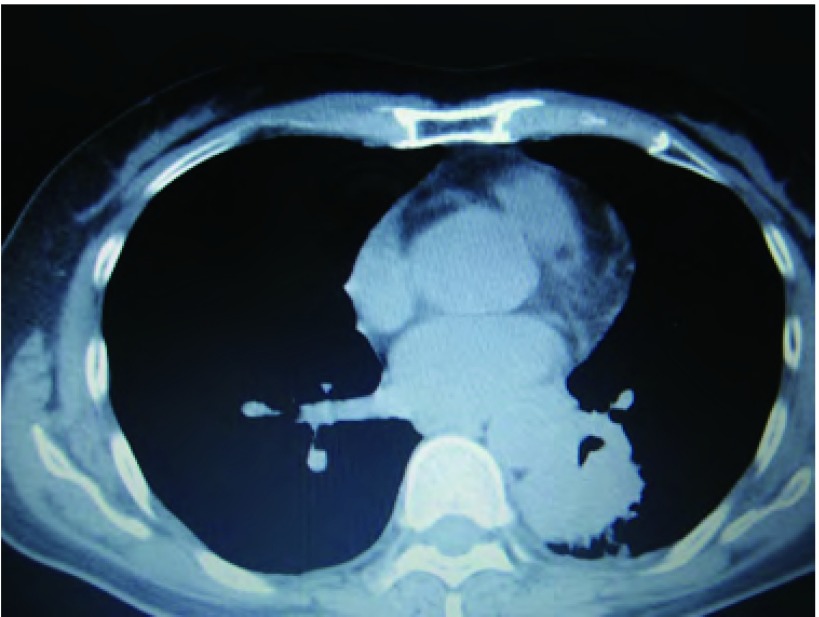
患者临床影像学特征。胸CT示左下肺中央型肺癌。 Clinical radiologic features of the patient. The CT of chest showing left lower lung central type lung cancer.

**2:**
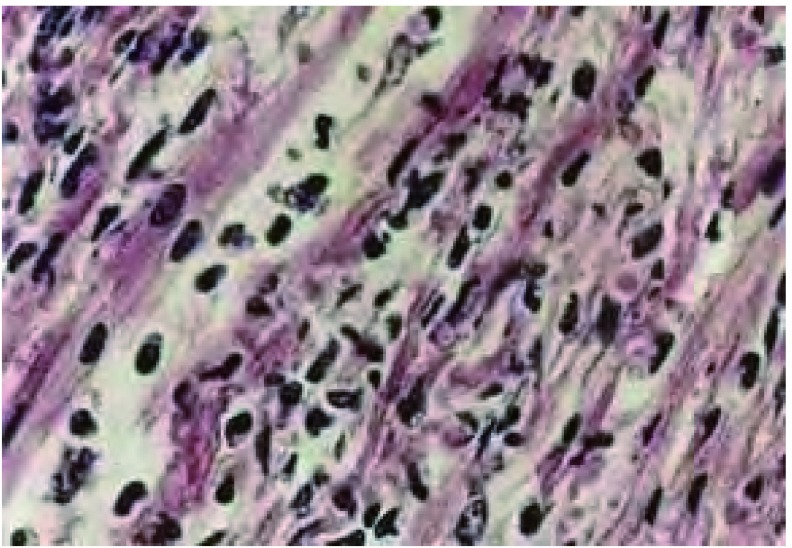
肌炎组织病理切片 The patient's myositis pathological section

## 2 讨论

肺癌细胞在增生分化过程中会分泌一些特殊的激素、抗原、酶等物质，使患者出现如低钙血症、高钙血症、肌无力综合征、多发性肌炎、周围神经炎等，表现为抽筋、肌肉痛、手麻、杵状指（即手指端脚趾端肿大）、全身无力等征象。多发性肌炎和皮肌炎是横纹肌非化脓性炎性肌病，其临床特点是以肢体近端肌、颈肌及咽肌等肌组织出现炎症、变性改变，导致对称性肌无力和一定程度的肌萎缩，并可累及多个系统和器官，亦可伴发肿瘤^[1]^。多发性肌炎（polymyositis, PM）指无皮肤损害的肌炎，伴皮疹的肌炎称皮肌炎（dermatomyositis, DM），该病属自身免疫性疾病，发病与病毒感染、免疫异常、遗传及肿瘤等因素有关。肌炎的诊断标准：①近端肌肉无力；②特征性皮疹；③血清激酶含量升高；④肌活检改变；⑤特殊的肌电图三联征。治疗上是急性患者给予强的松40 mg/d-60 mg/d或更大剂量，连续测定血清激酶活性为观察疗效的最好方法，大多数患者治疗后6周-12周内肌酶下降接近正常水平，随之肌力得到改善，肌酶含量一旦恢复正常，慢慢减小强的松剂量，反之肌酶升高就要加大剂量，成人通常10 mg/d-15 mg/d强的松长期维持，激素治疗无效患者可使用免疫抑制剂。本病例符合上述肌炎诊断依据，给予激素治疗同时胸部放射治疗肺部原发病灶，随访血清激酶有下降后出院至门诊随访。既往文献报道肺癌合并肌炎病例数不多，徐卫国等^[2]^报道以皮肌炎为首发症状肺癌1例，发病初以皮肤瘙痒症状明显，行肌肉活检确诊皮肌炎，予大剂量激素和免疫抑制剂治疗，短期内复发且逐渐加重后发现右下肺占位病变，行肿块活检诊断低分化腺癌，继续激素治疗同时联合化疗肺部病灶缩小，皮肌炎症状也明显缓解未有复发，皮损逐渐消退。徐文俊等^[3]^回顾性分析6年来收治的21例DM/PM合并恶性肿瘤患者的病史，结果DM/PM合并恶性肿瘤发生率为11.54%，以肺癌、肝癌为主；肿瘤得到控制的患者，DM/PM病情明显好转；DM/PM合并恶性肿瘤的1年死亡率为57.89%，主要死因是恶性肿瘤及其并发症，得出结论DM/PM易合并恶性肿瘤，其肿瘤类型与当地好发肿瘤一致，DM/PM的病情与恶性肿瘤的控制情况有密切关系，DM/PM合并恶性肿瘤患者的预后不良。陈小红等^[4]^回顾分析313例皮肌炎/多发性肌炎患者中45例（14.38%）伴发恶性肿瘤，伴发的肿瘤主要为鼻咽癌、肺癌、乳腺癌，69%的患者恶性肿瘤发现于皮肌炎的诊断时，伴发肿瘤的高危因子包括皮肌炎的发病年龄晚（> 40岁）及病程短（< 1年）。所以肿瘤相关肌炎/皮肌炎与神经系统副癌综合症的发病机制相似，治疗均以控制肿瘤为主且均可使用免疫治疗，皮肌炎患者需警惕恶性肿瘤，肿瘤可同时合并多种副癌综合征，因此不能只满足于一个诊断^[5]^。
